# The ethics of pet robots in dementia care settings: Care professionals’ and organisational leaders’ ethical intuitions

**DOI:** 10.3389/fpsyt.2023.1052889

**Published:** 2023-01-23

**Authors:** Wei Qi Koh, Tijs Vandemeulebroucke, Chris Gastmans, Rose Miranda, Lieve Van den Block

**Affiliations:** ^1^College of Nursing, Medicine, and Health Sciences, University of Galway, Galway, Ireland; ^2^Sustainable AI Lab, Institut für Wissenschaft und Ethik, University of Bonn, Bonn, Germany; ^3^Centre for Biomedical Ethics and Law, Faculty of Medicine, KU Leuven, Leuven, Belgium; ^4^End-of-Life Care Research Group, Vrije Universiteit Brussel (VUB) and Ghent University, Brussels, Belgium

**Keywords:** pet robots, social robots, ethics, ethics of aging, dementia, nursing homes, long-term care, implementation

## Abstract

**Background:**

Pet robots are gaining momentum as a technology-based intervention to support the psychosocial wellbeing of people with dementia. Current research suggests that they can reduce agitation, improve mood and social engagement. The implementation of pet robots in care for persons with dementia raises several ethical debates. However, there is a paucity of empirical evidence to uncover care providers’ ethical intuitions, defined as individuals’ fundamental moral knowledge that are not underpinned by any specific propositions.

**Objectives:**

Explore care professionals’ and organisational leaders’ ethical intuitions before and when implementing pet robots in nursing homes for routine dementia care.

**Materials and methods:**

We undertook a secondary qualitative analysis of data generated from in-depth, semi-structured interviews with 22 care professionals and organisational leaders from eight nursing homes in Ireland. Data were analysed using reflexive thematic analysis. Ethical constructs derived from a comprehensive review of argument-based ethics literature were used to guide the deductive coding of concepts. An inductive approach was used to generate open codes not falling within the pre-existing concepts.

**Findings:**

Ethical intuitions for implementing pet robots manifested at three levels: an (1) individual-relational, (2) organisational and (3) societal level. At the individual-relational level, ethical intuitions involved supporting the autonomy of residents and care providers, using the robots to alleviate residents’ social isolation, and the physical and psychosocial impacts associated with their use. Some care providers had differing sentiments about anthropomorphizing pet robots. At the organisational level, intuitions related to the use of pet robots to relieve care provision, changes to the organisational workflow, and varying extents of openness amongst care providers to use technological innovations. At the societal level, intuitions pertained conceptions of dementia care in nursing homes, and social justice relating to the affordability and availability of pet robots. Discrepancies between participants’ ethical intuitions and existing philosophical arguments were uncovered.

**Conclusion:**

Care professionals and organisational leaders had different opinions on how pet robots are or should be implemented for residents with dementia. Future research should consider involving care practitioners, people with dementia, and their family members in the ethics dialogue to support the sustainable, ethical use of pet robots in practice.

## Introduction

Worldwide, the number of people living with dementia continues to rise rapidly (1). Technological advancements have led to the rise of several supports and tools to meet the needs and care of people living with dementia, one of which are care robots. These broadly encompass physically assistive robots and social robots. Physically assistive robots are designed to provide a range of physical assistance to users with different mobility needs. Examples include robotic wheelchairs and feeding aids (2). In contrast, social robots are designed to support users through social interactions. These may be broadly classified into different categories based on their functions (3). First, socially assistive robots can support users with different tasks apart from facilitating social interactions. Examples include Pepper (4), a robot that can be programmed to engage people with dementia in cognitively stimulating activities (5), and MARIO (6), which provides nursing home residents with dementia access to personalised calendars and photos for reminiscence (7). Telepresence robots are non-autonomous or semi-autonomous robots that mostly comprise a videoconferencing monitor mounted on a mobile platform. These robots can be controlled remotely by an operator, such as care personnel or users’ family members. This enables the robots to move around the users’ home environment, which supports a sense of presence by the operator and the user (e.g., people with dementia). Examples include Giraff and Double, which have been used in long-term care facilities, such as nursing homes, to support social interactions between residents and their family members (8, 9). Finally, pet robots are designed to resemble animals in terms of their appearances and behaviours. Examples include Aibo (10), a robotic dog, Justocat, a robotic cat, and PARO (11), a robotic seal. Current evidence suggests that pet robots can elicit positive impacts on several psychosocial dimensions of people with dementia in long-term care settings, such as reduced agitation, improved mood and social interactions (12–14). Correspondingly in practice, pet robots continue to be implemented in care settings to support the social health of people with dementia (15). However, adopting pet robots for dementia care remains a contentious topic, as their ethical underpinnings are still heavily debated (16).

Based on a systematic review, Vandemeulebroucke (17) identified seven normative (i.e., argument-based) ethical domains relating to the use of care robots in the care of older adults (Table 1). While care robots encompass a broader characterisation than pet robots (i.e., care robots broadly include both physically assistive and social robots) and the identified ethical domains do not specifically relate to the context of dementia, these domains do represent a comprehensive overview of the existing ethical debates in the field. As such, they provide a good starting point to support a thorough consideration of ethical concerns relating to the use of pet robots. An overview of these domains and their relevance in the field of pet robots in dementia care is presented below.

**TABLE 1:** Ethical domains relating to the use of care robots.

Ethical domains	Brief description
1. Respect for autonomy and privacy	Impact of care robots on the autonomy and privacy of older people and their care providers
2. Dignity, objectification, and deception	Impact of care robots on the dignity of older adults, since machines cannot recognise them as persons. This could lead to feelings of objectification. Deception can occur when robots are designed to resemble living beings and become perceived as such
3. Replacement	Perceiving and regarding care robots as replacements for human caregivers
4. Social isolation and loneliness	The potential impact of care robots on older adults’ feelings of loneliness and social isolation, however, there could be potential negative impacts
5. Safety, physical and psychological harm	Potential of physical and psychological harm relating to the use of pet robots, such as distress
6. Social justice	Equal access and fair distribution of care robots, and the legal responsibility relating to their use
7. Conceptions of care	Impact of the use of care robots on the societal, organisational, and individual conceptions of what aged care entails and means. The societal values relating to the use of care robots for older people, such as the values of care provision

First, ethical issues relating to respect for autonomy and privacy are underlined. Autonomy is defined as “[…] the principle of self-determination when a person is allowed to make free choices about what happens to him or her, the freedom to act and decide, based on clear, sufficient and relevant information to participate in the decision-making” (18). While some arguments support the use of robots to preserve or empower users’ autonomy such as their sense of independence (19), concerns relate to their potential to infringe on users’ freedom and privacy (20).

The second domain relates to the impact of care robots on users’ dignity, the objectification of those who use care robots, and the act of deceiving users about the real nature of care robots. Some ethicists argue that robots entail deliberate deception through their design by realistically modelling the appearance and behaviours of pet robots after living pets, which can lead users to regard them as living beings (21–23). This has been argued to be deceitful, objectifying, and dismissive of users’ dignity (24). In practice, these arguments appear to manifest less strongly. In two qualitative studies, Moyle and colleagues found that while a few family members and long-term care staff expressed concerns about “infantilising” people with dementia through using pet robots, other participants appeared to place more weight on their positive impacts on users, such as improvements in mood (25, 26). Similarly, previous studies revealed that some people with dementia interacted with them as if they were real animals and received comfort from their use (27, 28). This seems to align more with other ethical arguments suggesting a focus on the consequences (i.e., positive or negative) of and motivations behind the “deception” (23).

The third domain relates to concerns that robots will replace human caregiving (17), especially with a rapid global ageing population and the insufficiency of available caregivers in long-term care settings (29). In such settings, care professionals have expressed similar fears that the use of robots could render their jobs obsolete and dehumanise care for people with dementia (30), however, such arguments relate more to socially assistive robots rather than pet robots.

The fourth domain relates to the potential of robots to alleviate social isolation and loneliness when older adults establish a bond with robots or leverage them as social facilitators (17). Studies have found that, when used in both group and individual settings, older people and people with dementia could form an emotional bond with pet robots which have led to positive benefits such as social engagement (12, 26, 27).

Nevertheless, as the fifth domain indicates, the use of care robots can also cause negative repercussions and corresponding concerns about the safety, physical, and psychological harm relating to their use, such as distress and over-reliance on robots. Moreover, while the use of robots can have positive benefits in alleviating social isolation and loneliness, some studies also reported issues stemming from the attachment to pet robots, such as jealousy or possessiveness (31, 32).

The sixth domain relates to social justice, grounded in the notion that all individuals should have equitable access to rights and opportunities (33). The literature has shown that the cost of pet robots, particularly PARO, which costs approximately 6,000 euros per unit, is prohibitive (25, 34). Lower-cost and less technologically nuanced options, such as the Joy for All (JfA) pets (approximately 110–130 euros each) and Tombot, a robotic dog, have emerged in recent years. Their impact on people with dementia appears to resemble other more technologically sophisticated robots (12), and their lower cost appears to have accelerated their adoption in practice. For instance, over 20,000 JfA pets have been distributed in the United States to support the social well-being of older adults (35). Similarly, many residential facilities in Sweden have adopted the JfA pets due to their relative affordability (36).

The seventh domain, conceptions of care refer to the impact of care robots on the societal, organisational, and individual conceptions of what care for older adults entails and means. Moreover, the different societal values related to these possible different conceptions, have been debated in the literature.

While there is a significant amount of normative ethics literature and commentaries on potential ethical issues of the use of care robots, including pet robots, in the care for older adults and specifically those with dementia, there is limited empirical research that has explored the ethical intuitions of care providers in nursing homes. We characterise ethical intuitions as individuals’ fundamental moral knowledge that is not underpinned by any specific propositions (37). Stahl and Coeckelbergh (38) argue that ethical arguments are often not congruent with care practices in real-world settings and that there has been insufficient consideration of care providers’ intuitions. With a growing interest in pet robots and their uptake in long-term care settings to support residents with dementia, it is important to bridge the gap in the literature by understanding the ethical intuitions of care professionals (CPs) and organisational leaders (OLs) in long-term care facilities. The purpose of this study is to explore the ethical intuitions before and when implementing pet robots in nursing homes for routine dementia care, from the perspectives of care professionals and organisational leaders. The term “care provider” will be used interchangeably in this paper to refer to both care professionals and organisational leaders.

## Materials and methods

We conducted a qualitative secondary analysis (QSA), which involved the use of existing data to understand a research question that differs from that of the primary research (39; Table 2). In the dataset derived from the primary research, participants’ ethical intuitions relating to the implementation of pet robots for dementia care in nursing homes were observed.

**TABLE 2:** Research questions in primary research and current research.

Research question (Primary research)	Research question: Secondary analysis (Current study)
What are the multilevel determinants to implementing pet robots in nursing homes for dementia care, from the perspectives of healthcare professionals and organisational leaders?	What are the ethical intuitions before and when implementing pet robots in nursing homes for dementia care, from the perspectives of healthcare professionals and organisational leaders?

However, exploring ethical intuitions was not the focus of the original research. As such, a secondary analysis was considered appropriate to address this observed phenomenon (40). Based on Heaton’s classification of QSA, our approach aligns with a “supra analysis” approach, where the focus of the secondary questions transcends the focus of the original research to “examine new empirical, theoretical or methodological questions” (41). As the lead researcher (WQK) conducted the primary research, the risk of decontextualization during this secondary analysis was minimised.

### Original research study

The original dataset was derived from a descriptive qualitative study, which explored the determinants of implementing pet robots for nursing home residents with dementia (40).

### Sampling and data collection

Twelve care professionals and 10 organisational leaders from eight nursing homes in Ireland were recruited through maximum variation purposive sampling and snowball sampling. Twenty-two individual, in-depth semi-structured interviews were conducted between August and November 2021. The interview guide (Supplementary material 1) was built upon findings from a previous scoping review (3) and developed using the Consolidated Framework of Implementation Research (CFIR), an implementation framework that guided the comprehensive exploration of the determinants of implementation (42). This interview guide was piloted before data collection; WQK conducted two pilot interviews, iteratively adjusting the interview questions and prompts to improve their clarity and flow after each pilot before each interview, participants were shown a short 5-min video that demonstrated the functions and features of two pet robots: the Joy for All robotic cats and PARO. In this video, WQK introduced the features and functions of each robotic pet. This included information about their dimensions, how they respond to users, how they are operated, charging and warranty, their cost, and general care and handling. Each interview lasted between 31 and 54 min. All interviews were audio recorded and transcribed verbatim. Detailed methods are described elsewhere (40, 43).

Table 3 shows a summary of the participants’ demographics. Most were female (*n* = 18) and the majority had over 7 years of experience in dementia care (*n* = 14). While over half (*n* = 13) had seen a pet robot before the interviews, only one-third of the participants (*n* = 7) had experience using them with residents with dementia.

**TABLE 3:** Participants’ demographic information.

	Sample
**Role**	
Care professionals	12
Nurse	5
Healthcare assistant	1
Activity coordinator	2
Occupational therapist	3
Physiotherapist	1
Organisational leaders	10
Assistant/Director of nursing	6
Assistant/Clinical nurse manager	3
Occupational therapy manager	1
**Age group**	
20–29 years old	2
30–39 years old	5
40–49 years old	10
50–59 years old	3
> 70 years old	1
No information	1
**Length of experience in dementia care**	
<1 year	1
1–3 years	3
4–6 years	3
7–9 years	3
>10 years	11
No information	1
**Experience with pet robots**	
**Seen a pet robot**	
Yes	13
No	9
**Used a pet robot**	
Yes	7
No	15

### Data analysis

Reflexive thematic analysis (RTA), as described by Braun and Clarke (44), was conducted. A combination of inductive and deductive approaches was adopted. The ethical constructs that were derived from Vandemeulebroucke’s comprehensive review of normative ethics literature were used to guide the deductive coding of concepts that are conducive to addressing the research question (17). However, an inductive approach was also used to generate open codes that did not fall within the pre-existing concepts but were reflective of the nature of the data. First, although WQK was involved in the original research, she re-familiarised herself with the anonymised data by reading and re-reading it again, annotating the data based on her impressions. Next, WQK recursively coded the data. In the first iteration, pre-defined codes and preliminary open codes were assigned to the data. This went through a few iterations, involving back and forth steps of moving between the transcripts and the codes, and revising the codes between iterations (45). Between these iterations, the research team had two meetings to explore different perspectives on the data and codes. This enabled us to leverage our multidisciplinary expertise (occupational therapy, biomedical and care ethics, psychology, nursing) to achieve richer interpretations of the meaning of the data (44). Afterward, our focus shifted to interpreting the meaning of the data as a whole. WQK organised the codes into candidate themes and subthemes, reinspected the codes within each theme for coherence, and considered identified patterns within the data. This led to the recoding and revision of themes. Finally, the themes were named and presented. Using a computer-assisted data analysis software for data management (NVivo12) allowed for a clear audit trail, which enhances dependability (46). The involvement of a multidisciplinary team with various expertise in the data analysis processes enhances the credibility of our findings (47).

### Reflexivity

A pragmatist ontology and epistemology, which places an overarching emphasis on experiential and actionable knowledge (48), underpinned both the primary research and this secondary research. In terms of personal reflexivity, the lead researcher (WQK) is an occupational therapist with clinical experience working with people living with dementia in different care contexts. In analysing the data, WQK adopted both an etic and emic positionality by leveraging her experiences to understand the “grassroots” perspective of implementing pet robots for dementia care in real-world settings, focused on understanding the practical and action-focused aspects of participants’ sentiments. These reflexive thoughts were annotated throughout the process of data analysis, and discussed with the multidisciplinary team to support a more holistic analysis of the data.

### Ethical considerations

The original study received ethical approval from the University of Galway (Ref no: 2020.10.014), where participants provided informed consent. Since the secondary research question is directly related to the intention of the primary research to explore the implementation of pet robots, consent gained in the primary research is considered sufficient for this secondary analysis (49, 50). All data for the QSA was fully de-identified.

## Findings

**FIGURE 1:**
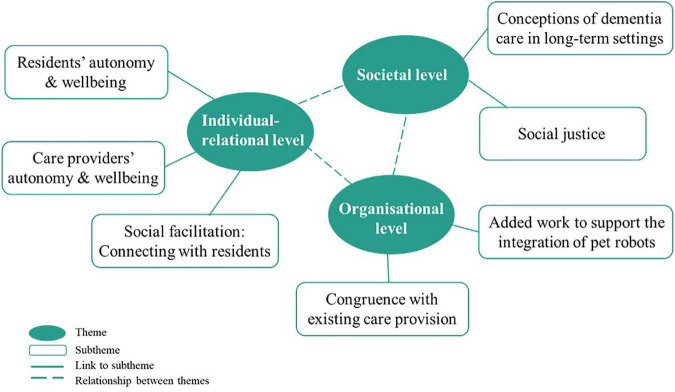
Thematic map of key themes structured on three levels.

Seven themes structured according to three levels were generated. The ethical intuitions of care professionals and organisational leaders occurred on different levels in the implementation of pet robots in nursing homes for residents with dementia: on an (i) individual-relational level, (ii) organisational level, and (iii) societal level. Figure 1 shows the thematic map.

## Individual-relational level

### Theme 1: Residents’ autonomy and wellbeing

Both care professionals and organisational leaders were unanimous in describing the importance of aligning pet robots with residents’ preferences by considering their life history, whether they liked animals, and the type of animals that they liked. Most described the pet robots as “realistic-looking,” and some further elaborated on the importance of their appearance for residents’ acceptance of the device:

“… *if you really want it to work, it has to look like the animals that you’re trying to mimic*…. *I think the visual is extremely important, especially for somebody with dementia [CP4].”*

Participants who have used pet robots described their ability to mediate social isolation and loneliness by providing a sense of companionship, and to support the psychosocial health of residents by supporting them to engage in activities, providing calming effects and improving their mood:

“… *he is happy you know*… *I think it (pet robot) has that impact on his quality of life and for some other ones (residents) as well. When they see the cat as well they smile, and they are happy [CP9].”*

Such intuitions were also shared by participants without experience in using pet robots, who described their potential to improve the psychosocial wellbeing of residents with dementia. Participants with experiences of using pet robots shared instances where residents became attached to the pet robots. Nevertheless, most merely described these circumstances and how they were managed, without attaching negative connotations to these situations. For instance, one participant mentioned how staff managed a situation where a resident’s daily routine was disrupted due to her attachment to the robot. Another described the nursing home’s response to a resident’s reluctance to share it with others:

“… *there’s even another couple of ladies (other residents) who think that it’s a real one and actually want to hold it. But now she’s become so possessive of it (pet robot) that she won’t let go to anybody else. So they (nursing home staff) are trying to get more (robotic) pet animals [CP11].”*

Most participants raised concerns about pet robots’ potential to cause physical harm such as the potential risk of falls and physiological harm through transmitting infections from one resident to another, particularly if they were shared between different residents. Two participants shared:

“… *it was more of a (fall) risk for her because she’d get up (to go to the kitchen to get milk for the robotic pet) and she’s not able to go on her own (due to mobility difficulties). But she’d be getting up and she kind of was getting irritated when you wouldn’t let her go to the kitchen to get the milk you know [CP7].”*

“… *if they’re carrying it (pet robot), is it a fall hazard [OL9].”*

### Theme 2: Care providers’ autonomy and wellbeing

Most participants with and without experience with pet robots described their ability to support and improve the wellbeing of residents as their main value and expressed openness to using technological innovations such as pet robots. Participants also described a sense of satisfaction from seeing residents’ responses to these robots:

*“I suppose it’s a feelgood factor to it that you kind of say “ah God isn’t that sweet.” It’s nice to see him happy and it gives him so much joy and I suppose that has a knock-on effect on us [CP8].”*

A few described fear or dislike of real animals, or potential difficulties working in a nursing home with real pets. Correspondingly, robots are considered a welcome alternative:

*“Now they have a lot of live cats up in that nursing home*… *I wouldn’t be able to work in it because I’m petrified of cats whereas the little fellas here (pet robot), I don’t mind them [CP7].”*

Participants with experiences using pet robots did not report concerns about whether residents treated pet robots as real; in fact, some expressed that this was beneficial:

*“Even though they can’t express it. If they think it’s real it has to have a beneficial physical and mental benefit to them*… *I would think anyway [CP8].”*

Similarly, some participants without experience expressed concerns that if used with residents without cognitive difficulties, pet robots may be perceived as infantilising. One care professional expressed that residents’ misperception of pet robots as real animals may lead them to experience distress, emphasizing that they should not be humanised:

“… *you don’t actually want somebody*… *associating with it as if it was a real animal because that could cause further distress down the line if they feel, well, I’ve never seen it eating or it doesn’t seem to be drinking*… *they could be stressors to the person with dementia*… *I think in so far as is possible you’d want to be introducing it that it is a robot [CP10].”*

### Theme 3: Social facilitation and connecting with residents

Participants with experience of using pet robots described using the robots to connect and communicate with residents, which may otherwise be difficult:

“… *she has very limited language skills at the moment*… *so if you ask her “how does the cat feel today?” and she’ll say “yeah, good.” in relation to the cat she can talk about it. But if you ask her any other question you may not get an answer at all [CP11].”*

Others also shared about using the robots to support residents to engage in routine care, such as using pet robots to motivate them to get out of bed or to take their medication. When conversing with residents about the pet robots, participants appeared to go along with their reality, speaking about the robots as if they were real animals:

“… *we will say it to our residents “is little kitten okay here in the basket or is he okay here.” if that’s their reality and they believe the cat is real. they’re happy with that [CP5].”*

Participants without experience had similar intuitions about the potential of using the pet robots as social facilitators.

## Organisational level

### Theme 4: Congruence with existing care provision

Both care professionals and organisational leaders expressed that certain aspects of implementing pet robots for dementia care aligned with the existing care routine within their nursing home. This included existing knowledge of residents’ likes and dislikes, which allowed participants to have an idea of each resident’s suitability for pet robots; having dedicated time for activities that have been (or can be) used to introduce pet robots to residents; and having skilled staff who are equipped to introduce new interventions such as pet robots. Some further described that the use of pet robots may be less effortful than other existing interventions since they require lesser logistics and planning:

*“Now it would be harder to get time for other activities like if we were to start a big painting session or gardening or baking that would be really, really hard because we just haven’t enough people. But the beauty of robot cuddly toy therapy is it really does itself [CP5].”*

Some participants described pet robots as replacements for real animals, since the former required lesser maintenance than the latter. Although volunteers or family members sometimes brought their pets to nursing homes to visit residents, such visits were prohibited during times of COVID-19. In this sense, most considered pet robots as viable substitutes. However, a few participants expressed preferences for real animals, stating that they cannot be replaced with robots, describing the former as being more tangible:

“… *I’d much prefer to be able to have live animal input*… *There was one gentleman we had*…. *I saw him one day and he was feeding half a banana to a wild cat outside and the cat had come over and was eating it*…. *it actually showed me that this man made a connection, and he made a connection with nobody*… *and he wouldn’t have had any connection with the robot or a toy. He just wouldn’t have*… *[OL10].”*

Most with experience using pet robots expressed that they provided some form of relief during care provision by providing residents with companionship and by reducing challenging behaviours. Participants without experiences also anticipated similar impacts:

“*I think it (my work) will actually improve*… *it’s always (about) making them comfortable. Once they’re comfortable, your work’s easier [CP1].”*

A few participants added that since they are not able to attend to residents all the time, the use of pet robots may be able to “… *complement the whole feeling of a presence*…*[OL9].”* Only two participants briefly raised privacy concerns, but quickly dismissed them as they felt that the pet robots do not store personal information.

### Theme 5: Added work to support the integration of pet robots

Most participants who have used pet robots did not describe additional workflows for incorporating them into dementia care. However, a few organisational leaders and care professionals shared that staff in their nursing home had to put in the effort to gradually cease the use of pet robots with residents when their attachment to the robot impacted their daily routines. This involved discussions and decision-making with the care team. Participants with and without experiences with pet robots expressed the importance of assessing each resident’s suitability to interact with pet robots to mediate potential distress.

“… *it’s not a one size fits all, either. Really, this should be an assessment criteria [OL3].”*

Those without experiences reported more anticipated additions to their work routine. This included identifying staff who are responsible for maintaining the pet robots, such as ensuring that their batteries are charged or replaced and maintaining their hygiene. One participant added that this may cause a burden for care staff:

“… *(this will cause) extra burden to the staff*… *anything you bring in. someone has to be responsible. This will be an extra task for the staff [OL6].”*

## Societal level

### Theme 6: Social justice

The cost of pet robots, especially PARO, was raised as a concern by the majority of participants. Some care professionals and organisational leaders shared that they were unclear about whether residents, their family members, or the nursing home should be responsible for purchasing pet robots for residents. Many positioned pet robots within a bigger picture of dementia care provision. Some participants expressed that residents often have financial constraints, and financial resources are prioritised to support the cost of basic nursing care. In this sense, pet robots may be considered unaffordable:

“… *those that are living in the nursing home. spend a lot of money to maintain their care. their pension goes into the confines of the HSE (Health Service Executive). Many of them have to sell their properties*… *to make sure that they get the care that they need. it would be difficult for their families to be asked for another huge amount of money like €6000 [CP9].”*

Similar constraints were described at the institutional level, where participants described difficulties acquiring or dedicating funds to purchase the robots:

*“We all work with budgets. what if you don’t have the money and the budget for it, well then you can’t buy it [CP4].”*

Some also expressed concerns about the cost-effectiveness of pet robots, considering that not all residents may like them or may lose interest in them over time. In contrast, participants described the JfA cat as being more affordable, which provides the opportunity for individualised use. Many emphasised the importance of individualising pet robots for residents since pets are often seen as “individual properties” and shared use may lead to issues such as jealousy or infection transmission. However, cost issues were described to prohibit (or will prohibit) individualised pet robot use.

### Theme 7: Conceptions of dementia care in long-term care settings

Based on personal experiences as pet owners or through seeing residents’ responses to pets, many participants expressed convictions about the role of pets in supporting residents:

*“I’m not a pet lover, okay, that’s personally me, but like you know, I really do think that pets do make a difference in the residents’ lives [CP11].”*

Some expressed that more nursing homes are becoming amenable to keeping real animals or allowing pet visits. While many were receptive to the use of robotic alternatives in dementia care, a few maintained their preferences for real animals:

*“there’s nothing like a live animal really. and you would know the people who are responsive to that. So I suppose I’d prefer to see much more of the input from therapy dogs before I would ever think about getting you know, something like Paro [OL10].”*

Next, many participants expressed the importance of upholding individualised and tailored dementia care within their nursing homes. This concept also applies to the use of pet robots, as many expressed that pet robots are not a one-size-fits-all solution, and anticipated that the need to facilitate the use of pet robots differently with each resident since they have different preferences and functional abilities. These were often described in relation to regulatory authorities’ quality indicators for care provision:

“… *(in) the guidelines with HIQA (Health Information and Quality Authority), we’re supposed to be providing activities to people*… *and (pet robots) would be very good activities. It’s individualised because if the person that likes it he would have it, and if the person that doesn’t want to engage. that’s fine [CP8].”*

## Discussion

In this study, we explored care professionals’ and organisational leaders’ ethical intuitions before and when implementing pet robots in nursing homes as part of routine dementia care, thereby making a contribution to limited empirical ethics literature in this field. It is evident that in this study there was a large overlap between care professionals’ and organisational leaders’ ethical intuitions. The use of existing normative ethical domains to support the data analysis process allowed for a comprehensive exploration of the study phenomenon. Except for privacy concerns, care providers’ (i.e., care professionals and organisational leaders) ethical intuitions manifested in all other ethical domains. Seven themes were generated, uncovering ethical intuitions at three levels (the individual-relational, organisational, and societal levels). While some align with existing normative ethical domains, findings illustrate several discrepancies which will be described below. A summary is shown in Table 4.

**TABLE 4:** Comparing ethical arguments with participants’ ethical intuitions.

Ethical domains	Existing ethical arguments	Ethical intuitions of care providers
1. Privacy and autonomy	• Impact of care robots on the autonomy and privacy of older people and their care providers	• Privacy concerns were not salient.
		• Pet robots empowered (or can empower) residents and care providers
2. Dignity, objectification and deception	• Impact of care robots on the dignity of older adults, since machines cannot recognise them as persons. This could lead to feelings of objectification. Deception can occur when robots are designed to resemble living beings and become perceived as such	• The realisticness of pet robots was viewed positively (support residents’ acceptance)
		• Most were in support of “going with residents” reality of treating pet robots as real animals
		• Few were concerned that residents’ misperceptions of them as real animals may lead residents to experience distress
3. Replacement	• Perceiving and regarding care robots as replacements for human caregivers	• Pet robots alleviated some aspects of caregiving
		• May be a viable substitute to real pets (e.g., pet visits)
		• Additional work (and workflows) may need to be in place to use pet robots in dementia care
4. Social isolation and loneliness	• The potential impact of care robots on older adults’ feelings of loneliness and social isolation, however, there could be potential negative impacts	• Pet robots provided (or had the potential to provide) companionship to residents
5. Safety, physical and psychological harm	Potential of physical and psychological harm relating to the use of pet robots, such as distress	• Potential physiological and physical harm (infection transmission, falls risk)
		• Participants reported issues with use (e.g., attachment and jealousy) but did not attach negative connotations to them. Rather, the focus was on risk assessment and managing situations
6. Social justice	• Equal access and fair distribution of care robots, and the legal responsibility relating to their use	• Affordability and availability of pet robots (particularly for individualised use)
7. Conceptions of care	• Impact of the use of care robots on the societal, organisational, and individual conceptions of what aged care entails and means. The societal values relating to the use of care robots for older people, such as the values of care provision	• Need to provide person-centred care, prioritisation of residents’ wellbeing
		• Regulatory authorities’ guidelines or requirements on care quality in nursing homes can influence the adoption of pet robots for dementia care
8. Psychosocial impacts*		• Pet robots can elicit (or have the potential to elicit) positive psychosocial impacts on residents and care providers

*New domain derived during the data analysis process.

### Inflated emphasis on deception and potential harm

The relationship between people with dementia and their formal caregivers is an important aspect of person-centred care (51, 52). However, establishing such relationships may be challenging for care staff in long-term care (51), particularly when residents exhibit challenging behaviours (53). In this study, pet robots appeared to serve as a medium for participants to communicate with and relate to residents. This often entailed going along with residents’ reality and engaging in conversations that appear to suggest pet robots as real animals. Participants also described the actual or anticipated potential of robots to enhance residents’ social health. Pet robots’ realisticness was perceived as an advantage to support residents’ interactions with (and benefits from) using the robots. These ethical intuitions clearly contrast existing ethical arguments which have placed a strong emphasis on the negative connotations of “deception” when the appearance of pet robots led users to believe pet robots are real (21, 22), so undermining users’ dignity (24). Rather than focusing on the act of “deception,” participants placed more focus on the impacts of pet robots on residents’ wellbeing. This echoes Bradwell and colleagues’ findings that younger adults also placed less emphasis on this ethical domain when considering the use of companion robots for older adults (54).

Another stark contrast between existing ethical arguments and care providers’ ethical intuitions relates to the potential harm from using pet robots. While the former cautioned about potential harm from using pet robots, such as distress and attachment, care providers in this study appeared to adopt a more practical-oriented approach involving risk assessments, tailoring and monitoring their use, and actively navigating “challenges” through discussions with the care team. In addition, residents’ “attachment” to pet robots was not considered an issue unless it negatively influenced residents’ well-being. Other studies have shown that some individuals with dementia have expressed desires to establish an emotional connection with pet robots by interacting with or caring for them (31, 55), even with the awareness that they were not real animals (28)–this has been described as voluntary “suspensions of disbelief” (19).

Pet robot interventions may have some parallels with doll therapy since both interventions appear to adopt similar mechanisms of therapeutic change by eliciting nurturing behaviours in people with dementia, who may also view and treat dolls as real babies. Attachment through doll therapy appears to be viewed in a positive light as it can address unmet needs (56–58). In this sense, attachment has been argued to be necessary for the self-preservation (e.g., seeking safety or relieving stress) of people with dementia, especially during instances of vulnerability (57). The central ethical orientation is on a “rights-based approach,” where the personal choices of people with dementia should be upheld wherever possible, and care professionals should respect and protect the needs and rights of these individuals (59). This ethical orientation appears to be endorsed in dementia care (59, 60).

### Imbalanced ethical focus on people with dementia

The existing ethical arguments have been heavily focused on the impact of pet robots on older adults and people with dementia. Although care providers play an important role in supporting people with dementia to use pet robots within long-term care facilities, current conceptions of their direct and indirect impacts on these stakeholders are somewhat overlooked. To illustrate, robots are developed based on the notion that there will be fewer caregivers to support the care of the rising population of older people. Additionally, ethical arguments outline concerns that pet robots may replace human caregiving or dehumanise care. These views suggest that technological solutions for dementia caregiving are inevitable. However, as dementia care provision in long-term care facilities is complex, such views appear to be insufficiently nuanced. While pet robots may support care providers to provide care for people with dementia, they could also lead to additional burden and workload.

Congruent with the argument that pet robots “may replace caregiving,” care providers in this study expressed that pet robots eased or were anticipated to ease some aspects of caregiving. They also appeared to empower the autonomy of staff by allowing them to connect to residents, providing them with satisfaction when residents appear happier. This corresponds with findings from other studies demonstrating that staff satisfaction is influenced by residents’ satisfaction (61, 62). However, other influences on caregiving, such as additional work that may be needed to incorporate pet robots into dementia care, appear to be insufficiently considered within this argument. Responses of residents with dementia to pet robots can vary from person to person; furthermore, their responses can fluctuate over time and depend on context (31). Participants expressed similar intuitions and emphasised the need to assess, facilitate, and re-evaluate the suitability of pet robots for residents. They also shared intuitions about other potential additions to their workflow, such as team discussions, developing cleaning processes, and assigning staff with designated roles to take responsibility for the robots. Unlike privacy concerns relating to the use of socially assistive robots and telepresence robots (63), such concerns were not salient in this study. This is likely because pet robots do not record or store personal data in their current form, or because this is not the immediate or primary concern of care providers reflecting on implementation in practice.

### Accessibility and availability of pet robots

Participants repeatedly expressed that the unaffordability of pet robots (particularly PARO) prohibited their accessibility. This was also positioned within a broader picture, as participants described difficulties allocating a significant amount of money for the robots amidst other financial liabilities necessary for supporting long-term care. Similar concerns have also been raised in other studies (25, 34). It is noteworthy to mention that such concerns stem from research conducted in high-income countries such as Australia, the Netherlands, and Taiwan (25, 34, 64). However, approximately two-thirds of people with dementia worldwide live in low- and middle-income countries (65). As such, the accessibility of such therapeutic devices is likely to be even more limited. In addition, while the use of pet robots appears to be shared among residents (12), participants appear to favour the individualised use of pet robots for person-centredness and pragmatic reasons relating to infection prevention. For these reasons, it is unsurprising that most favoured the JfA cat, despite it being significantly less technologically sophisticated than PARO. The former is commercially available, and the commercial aspects may contribute to the lower price tag (63). Nevertheless, Ienca and colleagues (63) posited that the focus on the commercial aspects could reduce the level of safeguards, and disposing technological devices to legal, social, and technical risks. For instance, PARO can only be purchased through retailers and is regulated as a medical device by the Food and Drug Administration (FDA) in the United States, to ensure their safety for public use. In contrast, commercial alternatives like JfA pets are not regulated, and their use is at users’ discretion. Therefore, when choosing (lower cost) commercially available pet robots, researchers and care organisations must consider and weigh the cost of pet robots with potential risks.

### Implications for research and practice

Our findings revealed similarities and discrepancies between ethical arguments and care providers’ ethical intuitions, which suggest that ethical arguments may not always reflect ethical challenges or considerations in real-world settings, where there are more complexities to consider at various levels. At the individual-relational level, findings suggest that pet robots may impact residents and staff by supporting their autonomy, wellbeing, and relationship-building. Additionally, to mitigate potential harm, it may be necessary to assess the appropriateness of pet robots for individual residents. At an organisational level, while pet robots may alleviate caregiving, they may also increase the caregiving load. As such, it may be necessary to carefully consider care providers’ and organisations’ capacities to implement pet robots. At the societal level, while lower-cost pet robots could promote equal access, it may be necessary to balance cost with potential risks (66). Moving forward, it is necessary to advance the existing ethical arguments by involving care practitioners, people with dementia, and their family members in the ethics dialogue to support the sustainable, ethical use of pet robots in practice. After all, the use of these robots directly implicates these stakeholders.

### Strengths and limitations

Given the scarcity of literature that has explored the care providers’ ethical intuitions of implementing pet robots for dementia care in nursing homes, this study addresses a critical gap in the literature. Using a robustly developed ethical framework to guide the data analysis process supported the comprehensive considerations of participants’ ethical intuitions, allowing these ethical intuitions to be readily compared to the normative ethical literature. The process of data analysis was rigorous, had a clear audit trail, and followed established guidance (44). The involvement of a multidisciplinary team in the data analysis process also allowed for dialogue to support a holistic interpretation of the data. A key limitation is the conception of this study through a secondary analysis of existing qualitative data, where exploring care professionals’ and organisational leaders’ ethical intuitions were not the primary focus. This meant that we were not able to explore the phenomenon of interest in further depth and rather had to work within the confines of the primary dataset. Hinds and colleagues outlined the importance of researcher sensitivity to the context of the primary study during secondary analyses (39). This potential limitation is mitigated since the primary author conducted the original study and is well-familiarised with the original context and dataset. Next, participants in the study were purposely selected. Most participants appeared to express positive sentiments toward pet robots, and this may explain the discrepancies between their ethical intuitions and existing normative ethical reasonings. As such, their perspectives may not be representative of the intuitions of dementia care providers who may have less positive sentiments toward pet robots. Finally, participants were from Ireland, a high-income country where many own real pets. As such, findings from this study may not be reflective of other contexts, such as countries with different economic statuses and amenability to pets (67).

## Conclusion

This study uncovered care professionals’ and organisational leaders’ ethical intuitions of implementing pet robots for dementia care in nursing homes. The incongruences between their ethical intuitions and existing argument-based ethics literature are revealed, suggesting care providers had different opinions on how pet robots should, or are implemented for residents with dementia. As care providers, people with dementia, and their caregivers are directly implicated in the implementation of pet robots, they should be involved in future ethical debates and the development of ethical guidelines.

## Data availability statement

The data analysed in this study is subject to the following licenses/restrictions: The data generated and analysed in this study is not publicly available in order to maintain participant privacy and confidentiality. However, de-identified parts of the interview transcripts may be obtained from the corresponding author upon reasonable request. Requests to access these datasets should be directed to weiqi.koh@universityofgalway.ie.

## Ethics statement

The primary study was reviewed and approved by the University of Galway. All participants provided written informed consent to participate in the study.

## Author contributions

WK conceptualised and designed this study in consultation with TV, CG, RM, and LV, led the data analysis process, discussed the preliminary themes with TV, and wrote the first draft of the manuscript, with support from TV. TV, CG, RM, and LV were involved in discussing the coding iterations. All authors critically reviewed and/or contributed to the manuscript revision iterations and approved the final version for submission.
