# Supplementary material for: The ethics of pet robots in dementia care settings: Care professionals’ and organisational leaders’ ethical intuitions
Source: Front Psychiatry. 2023 Jan 23;14:1052889. doi: 10.3389/fpsyt.2023.1052889 (PMC9899814; doi:10.3389/fpsyt.2023.1052889)
Supplement: Supplementary file 1 [file Data_Sheet_1.pdf]

Additional File 1 - Interview Guide

1. What do you think about the two pet robots that you saw in the video?
2. Would you want to introduce pet robots into your workplace for residents with dementia? If yes, why (and if no, why not?)
3. What are some local or national policies/guidelines, if any, that has influenced dementia care in your workplace?
4. Do you know of other nursing homes of care organisations that have introduced pet robots for dementia care?
5. How do you feel about using pet robots as a part of your day-to-day work with residents with dementia?
6. What do you think would be required to introduce pet robots for residents with dementia in your workplace?
